# Single-shot Monitoring of Ultrafast Processes via X-ray Streaking at a Free Electron Laser

**DOI:** 10.1038/s41598-017-07069-z

**Published:** 2017-08-03

**Authors:** Michele Buzzi, Mikako Makita, Ludovic Howald, Armin Kleibert, Boris Vodungbo, Pablo Maldonado, Jörg Raabe, Nicolas Jaouen, Harald Redlin, Kai Tiedtke, Peter M. Oppeneer, Christian David, Frithjof Nolting, Jan Lüning

**Affiliations:** 1 Paul Scherrer Institut, 5232 Villigen PSI, Switzerland; 2 Laboratoire d’Optique Appliquée, ENSTA ParisTech - CNRS UMR 7639 École Polytechnique, Chemin de la Hunière, 91761 Palaiseau, France; 3 Sorbonne Universités, UPMC Univ. Paris 06, CNRS, LCPMR, 75005 Paris, France; 4 Department of Physics and Astronomy, Uppsala University, SE-75120 Uppsala, Sweden; 5 Synchrotron SOLEIL, L’Orme des Merisiers, Saint-Aubin, BP 48, 91192 Gif-sur-Yvette Cedex, France; 6 HASYLAB/DESY, Notkestrasse 85, 22607 Hamburg, Germany

## Abstract

The advent of x-ray free electron lasers has extended the unique capabilities of resonant x-ray spectroscopy techniques to ultrafast time scales. Here, we report on a novel experimental method that allows retrieving with a single x-ray pulse the time evolution of an ultrafast process, not only at a few discrete time delays, but continuously over an extended time window. We used a single x-ray pulse to resolve the laser-induced ultrafast demagnetisation dynamics in a thin cobalt film over a time window of about 1.6 ps with an excellent signal to noise ratio. From one representative single shot measurement we extract a spin relaxation time of (130 ± 30) fs with an average value, based on 193 single shot events of (113 ± 20) fs. These results are limited by the achieved experimental time resolution of 120 fs, and both values are in excellent agreement with previous results and theoretical modelling. More generally, this new experimental approach to ultrafast x-ray spectroscopy paves the way to the study of non-repetitive processes that cannot be investigated using traditional repetitive pump-probe schemes.

## Introduction

Ultrashort light pulses enable scientists to interrogate condensed matter on its fundamental time scales. With the advent of x-ray free electron lasers (XFELs), which produce extremely bright x-ray pulses as short as a few femtoseconds^1, 2^, the potential of x-ray based techniques has been extended to investigations on ultrafast time scale. This allows shining light on phenomena that are difficult to study using optical spectroscopy, as for example distinguishing the individual dynamics of different components in complex materials^3–6^. Typically, experiments resolving ultrafast processes rely on repetitive pump-probe techniques that do not allow probing of phenomena having a stochastic nature or systems that are difficult to reset repeatedly to the initial state. To overcome these limitations, various methods based on spatial and spectral encoding of the pump-probe time delay have been developed in optical spectroscopy, which allow for time reconstruction of an ultrafast process from a single optical laser pulse^7–9^.

Up to now, experiments employing single x-ray pulses from XFELs succeeded in capturing the transient state of a sample at a single time delay^10^, with the notable exceptions of multiple split and delay setups sampling a few discrete time delays at once^11, 12^. Here, we demonstrate a novel experimental method that makes it possible to continuously probe with a single femtosecond x-ray pulse the response of a system to an ultrafast excitation over an extended time interval.

**Figure 1:**
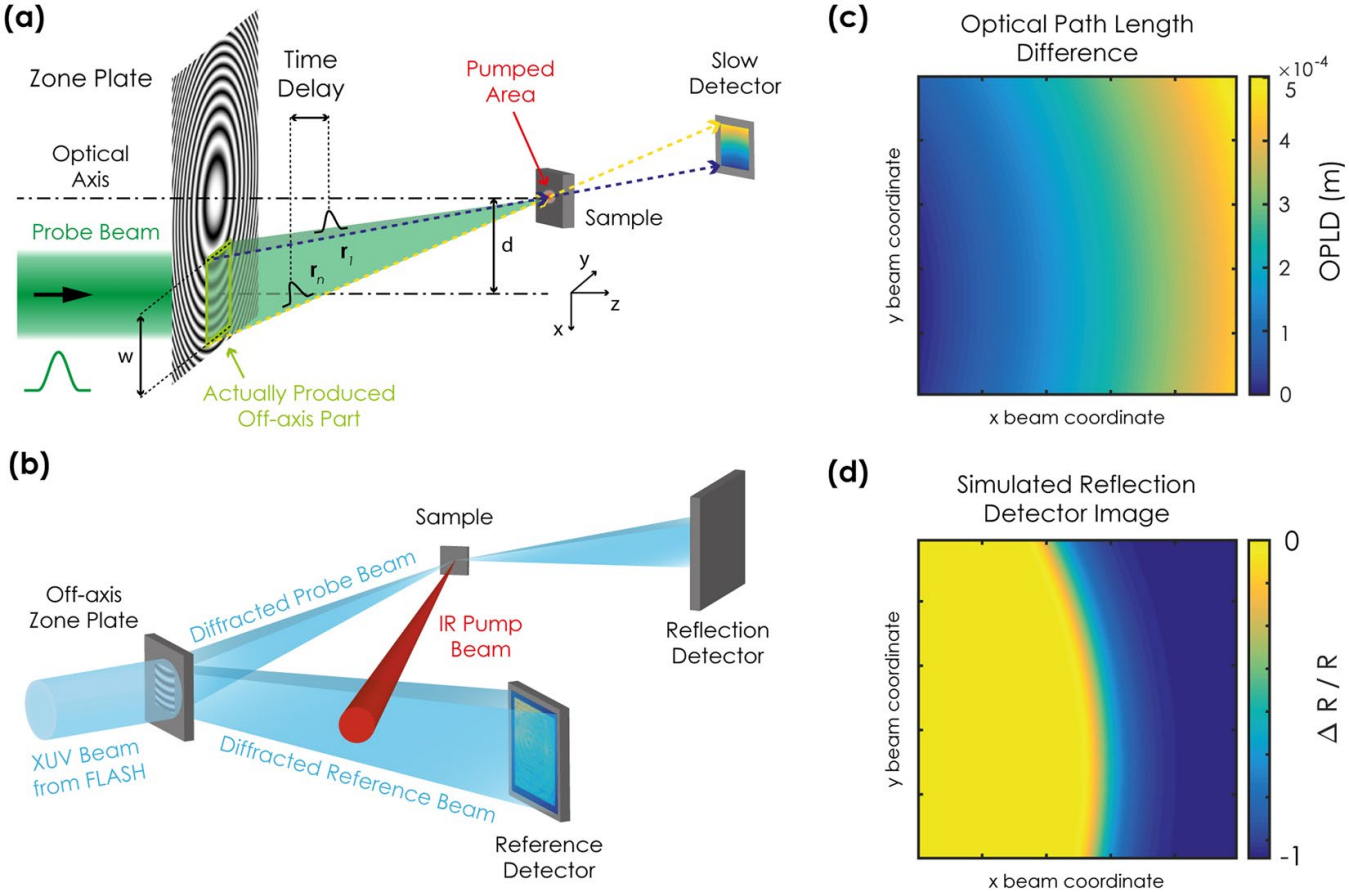
(**a**) Principle of the x-ray streaking technique, which is based on an off-axis Fresnel zone plate. Light travelling on rays closer to the zone plate optical axis probes the excited area on the sample earlier than light travelling along rays that are further away from it. After further propagation the rays separate again and each of them can be imaged on a slow detector, allowing for reconstruction of the ultrafast dynamics of the sample using a single x-ray pulse. (**b**) Schematic of the experimental setup. Details on the implementation are described in the text and in the Methods. (**c**) Calculation of the optical path length difference (OPLD) as a function of the beam coordinates of the reflected beam. (**d**) Simulation of the image recorded by the reflection detector in case of an ultrafast drop of the sample reflectivity caused by the IR pump pulse. The area in yellow (blue) corresponds to rays that arrive on the sample earlier (later) than the excitation pulse. The axes in (**c**) and (**d**) are rotated by 90° with respect to those in (**a**) and (**b**) such that time evolves primarily horizontally from the left to the right.

Our experimental technique, to which we refer to in the following as x-ray streaking, is based on a basic principle of diffractive zone plate optics. Each zone of the zone plate diffracts light to the focal point adding a delay of λ/c per zone, where λ is the wavelength of the x-ray pulses and c is the speed of light. This is illustrated in Fig. 1(a), the optical path length from a zone plate to its focal point is the shortest for rays diffracted from the innermost zone of the zone plate and it increases continuously with the radial distance from the zone plate centre. When a single probe pulse illuminates the zone plate, it is diffracted into a continuous set of sub-pulses that converge at the focus. As each sub-pulse propagates along a different path, each of them reaches the focus at a different time and with a different angle. Their propagation continues after the focus, and the sub-pulses separate again, reaching spatially distinct locations, e.g., on an area detector. In this way the arrival time of each sub-pulse to the focus is encoded into the spatial coordinates on the detector. When a sample is placed in the zone plate focus, these sub-pulses probe the sample at different times, e.g., with respect to the arrival of an external pump pulse exciting the sample. The time evolution of the sample properties is thus encoded in the image recorded by the area detector. The choice of an off-axis illumination of the zone plate^13^ is ideal to separate the different diffraction orders and to maximise the accessible time delay window for a given beam size.

X-ray streaking also overcomes a limitation of conventional pump-probe experiments at unseeded XFELs. The stochastic nature of self-amplified spontaneous emission results in strong fluctuations of x-ray pulse parameters such as arrival time or spectral composition^1, 14^. As conventional pump-probe measurements require averaging over many XFEL pulses, such fluctuations result, for example, in a deterioration of the achievable energy and time resolution. Retrieving the full time evolution of the ultrafast response of the sample using a single x-ray pulse avoids the need of averaging over a series of XFEL pulses, and allows approaching the achievable energy and time resolution given by the characteristics of a single XFEL pulse.

As the whole dynamics of an ultrafast process is captured in a single pump-probe event, x-ray streaking gives access to the ultrafast dynamics of stochastic phenomena and irreversible phase transitions in materials. Note that this is even useful when repetitive pump-probe measurements are possible, since capturing the entire trace at once allows selecting specific probe pulses, which can improve the data quality.

## Results

To demonstrate the capabilities of our novel x-ray streaking technique we investigated the ultrafast demagnetisation dynamics occurring in a thin ferromagnetic film upon non-thermal excitation by an intense, femtosecond short infrared pulse. This excitation causes the magnetisation of the sample to quench rapidly within the first picosecond^15, 16^. Unravelling the fundamental mechanism of ultrafast magnetization dynamics is expected to have strong impact on the understanding of magnetism and to lead possibly to novel technological applications^17–20^. Since up to now, all the experiments on ultrafast demagnetisation have relied on repetitive pump-probe techniques, it remains an open question whether this phenomenon is indeed governed only by a single reproducible mechanism, as commonly expected, or whether multiple pathways, characterised by different demagnetisation dynamics, are present.

To gain sensitivity to the transient magnetic properties of the sample we employed the resonantly enhanced transverse magneto-optic Kerr effect (T-MOKE)^21, 22^ in reflection geometry. The x-ray photon energy was tuned to the cobalt M_2,3_ edge (~60 eV, corresponding to 20.8 nm in wavelength) and the sample oriented close to its Brewster angle. In this experimental configuration a change in the sample magnetisation generates a variation in x-ray reflectivity of up to 40%. In Fig. 1(b) we show a sketch of the setup employed to record the transient changes in x-ray reflectivity using single x-ray pulses from the free electron laser FLASH. The unfocused FLASH pulse is used to illuminate the 4.8 × 4.8 mm^2^ Fresnel off-axis zone plate. The +1^st^ diffraction order beam is focused on the area of the sample excited by the infrared laser pulse. The sample reflects the beam towards a high sensitivity two-dimensional detector (reflection detector). The −1^st^ diffraction order beam is divergent and goes directly to a second two-dimensional detector (reference detector). We employ the reference detector to account for shot to shot fluctuations in the intensity profile of the XFEL beam, as well as inhomogeneities in the illumination and diffraction efficiencies of the zone plate.

We performed a ray optics calculation of how the optical path length from the zone plate to the sample varies as a function of the *x* and *y* coordinates of the 1^st^ order diffracted beam. Figure 1(c) shows the result of the calculation performed for the experimental geometry and the zone plate parameters used in this work (see the Methods section for more details). The maximum optical path difference is close to 470 μm corresponding to a maximum difference in arrival time of 1.57 ps. Using the time delay map retrieved from the ray-optics calculation we calculated how the image collected by the reflection detector would look in case of a sharp drop in reflectivity happening within 100 fs of the infrared excitation. The result shown in Fig. 1(d) indicates how the time delay between the pump pulse and the array of probe sub-pulses varies as a function of the spatial *x* and *y* coordinates of the image of the 1^st^ order diffracted beam. The expected radial symmetry is distorted due to the finite size (200 × 40 μm^2^) of the focal spot and the 45° incidence angle. This affects the optical path length of the rays and thus alters the spatial encoding of the pump-probe time delay in the reflection detector image.

**Figure 2:**
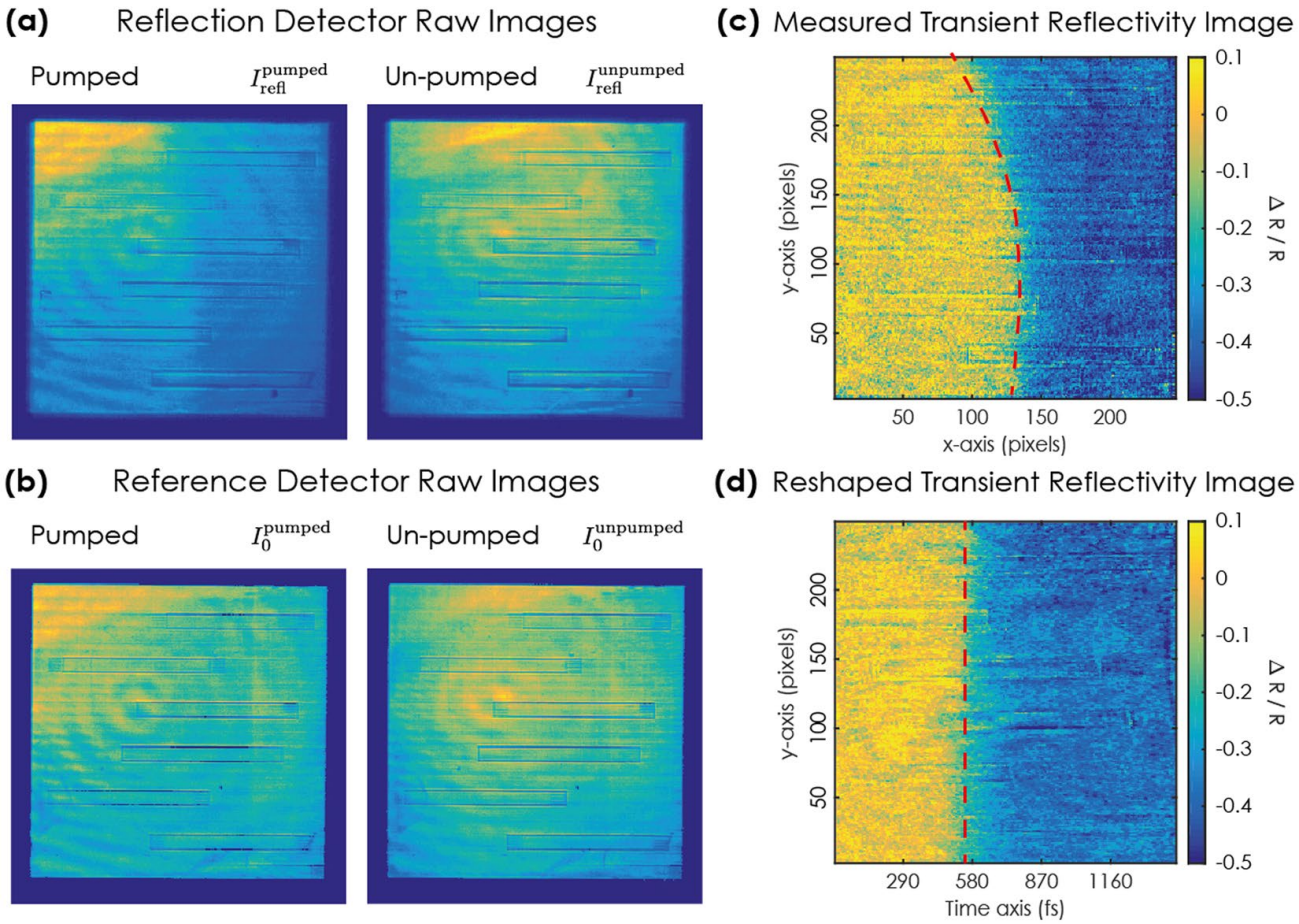
(**a,b**) Raw images from the reflection and reference detectors respectively. Both the images for the pumped and the un-pumped event are acquired using a single x-ray pulse. (**c**) Transient reflectivity image (as defined in the text) calculated from the images shown in (**a,b**). (**d**) Reshaped transient reflectivity image after calibration of the time window.

To experimentally quantify the transient magnetisation change induced by the infrared laser pulse, we collected reflectivity measurements using single XFEL pulses with (pumped) and without (unpumped) infrared excitation. The x-ray probe intensity was kept low enough not to alter the magnetic properties of the sample (<1 mJ/cm^2^, deposited at the sample over a time window of 1.5 ps), while the fluence of the infrared pump beam was set to about 15 mJ/cm^2^. The raw images acquired by the reflection detector using single x-ray pulses from the XFEL in the pumped (left) and un-pumped (right) cases are shown in Fig. 2(a). The expected signature of the demagnetization process is clearly visible in the raw image of the pumped measurement as an abrupt colour change from light to dark blue along the horizontal direction. This assignment is supported by the absence of this feature in the unpumped image. Hence, without the need of any data analysis these raw images already prove that the single-shot x-ray streaking method is working as expected.

Correcting for the inhomogeneous illumination and diffraction efficiency of the zone plate enhances the visibility of the transient change in reflectivity. For this we have derived the transient reflectivity image (TR-image) by pixel-wise computation of:

1 $$\frac{{\rm{\Delta }}R}{R}=\frac{{I}_{refl}^{pumped}/{I}_{0}^{pumped}}{{I}_{refl}^{unpumped}/{I}_{0}^{unpumped}}-1$$

where ${I}_{refl}^{pumped},{I}_{refl}^{unpumped}$, ${I}_{0}^{pumped},{I}_{0}^{unpumped}$ are the images collected by the reflection (Fig. 2a) and reference (Fig. 2b) detectors both in the pumped and unpumped case. The TR-image shown in Fig. 2(c) demonstrates that this procedure removes all illumination related artefacts. The colour scale is now dominated by the change in reflectivity due to the ultrafast demagnetization dynamics, which manifests itself along the x-axis as an abrupt change from bright yellow to dark blue. The red dashed line in the figure corresponds to a smoothed contour line of equal contrast change.

**Figure 3:**
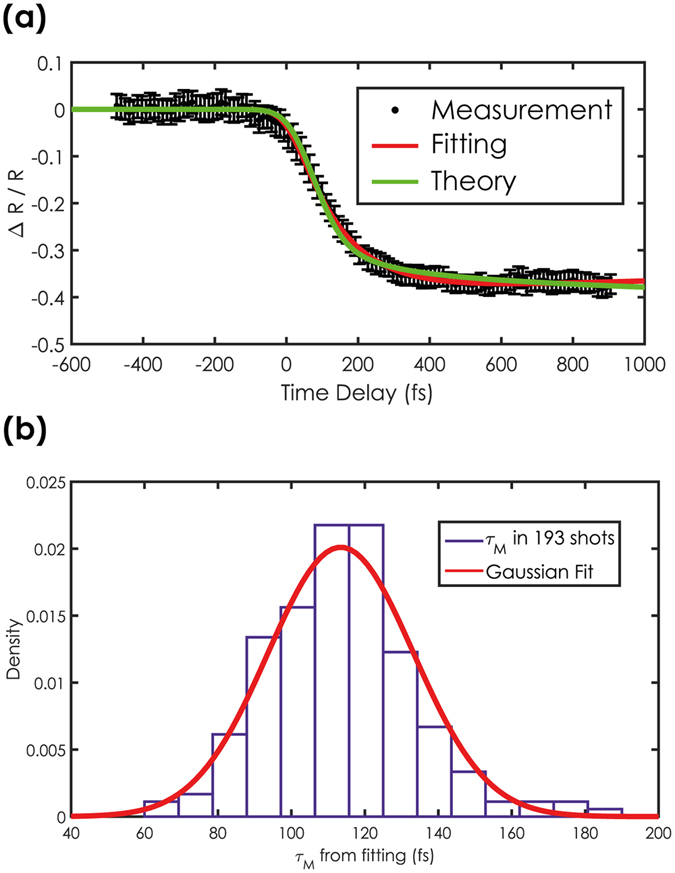
(**a**) Time resolved reflectivity curve extracted from the single-shot transient reflectivity image in Fig. 2(d). The red line represents a fit with a double exponential decay while the green line is obtained by calculations on the basis of super-diffusive transport theory. Error bars are the standard deviation calculated throughout the rows of the TR-image. (**b**) Statistical distribution of the demagnetisation times as extracted from the fitting of 193 single-shot measurements of the ultrafast demagnetisation process.

Knowledge of the delay map from the ray optics simulations allows us to extract the time resolved reflectivity curve from the transient reflectivity map. To do so, each row of the TR-image is interpolated onto a linearly varying time axis spanning between the earliest and latest time delay value common to all rows. Figure 2(d) shows the result of this linearization process. The abrupt contrast change is now a straight line (as highlighted by the red dashed line), i.e. time is varying linearly and only along the x-coordinate. By averaging the TR-image along the y-coordinate, one obtains the single-shot time resolved reflectivity curve shown in Fig. 3(a). We note that the signal-to-noise ratio of these single shot data is excellent, well comparable to what has been obtained so far by averaging over a large number of pump-probe events in repetitive pump-probe measurements^3^.

Upon infrared excitation, the reflectivity of the sample drops by approximately 40%. This corresponds to a complete demagnetisation of the cobalt layer as confirmed by static and repetitive pump-probe T-MOKE asymmetry measurements. The ultrafast drop in reflectivity, which characterizes the ultrafast magnetisation change, can be modelled using a double exponential decay from which one can extract the spin relaxation time τ_s_ (see Methods section for details). Fitting this model to the data yields the red solid line in Fig. 3(a), which determines the spin relaxation time τ_s_ to be (130 ± 30) fs, a value close to the experimental time resolution of ~120fs only given by the convolution of the duration of the pump and probe pulses (see Methods section for details). We note that this value is in general agreement with the results obtained on cobalt based alloys in other experiments using x-ray probe pulses^19^, confirming the soundness of this novel experimental approach. To further analyse the obtained data, we have theoretically modelled the ultrafast demagnetisation with the super-diffusive transport theory (see the Methods section for more details). The predicted magnetization evolution is represented in Fig. 3(a) by the green curve, which is in excellent agreement with the measured data, confirming the accuracy of the x-ray streaking method.

## Discussion

Being able to record an entire demagnetization curve with a single x-ray pulse opens up the possibility to follow the entire time evolution after each excitation event in a longer series of consecutive single shot experiments, thus providing novel insight into the underlying physical processes. Figure 3(b) shows the statistical distribution of the spin relaxation time τ_s_ obtained by fitting the time resolved reflectivity curves recorded for each of 193 consecutive events. The measurements follow a unimodal distribution, and the fitting of the experimental histogram with a Gaussian distribution shows an excellent agreement leading to a value of <τ_s_> of (113 ± 20) fs, in excellent agreement with the value obtained in a single pump-probe event. We note that this value is comparable with our experimental time resolution of ~120 fs and that within this limit, we do not observe a multimodal distribution peaked around more than one value that could indicate that the magnetic system followed different paths on each demagnetisation event. This observation illustrates that, within our time resolution, the evolution of the ultrafast demagnetization process is truly deterministic.

In general, the achievable time resolution with this x-ray streaking technique is limited by two factors. The first one is the duration of pump and probe pulses as in any other pump-probe experiment. In the data fitting in Fig. 3(a), we have set the time resolution to a value 120 fs, which corresponds to the convolution of the independently determined duration of pump and probe pulse. The result of the fit thus suggests that the time resolution in the experiment is essentially limited by the duration of the x-ray and infrared pulses. The second factor is related to the bandwidth of the incoming probe pulse, which plays an important role, since zone plates are energy dispersive optical elements. We calculated that in the particular geometry employed here, probe pulses with a bandwidth of 1.5%, as in our experiment, limits the maximum achievable time resolution to ~50 fs, a value lower than the duration of the pump and probe pulses we employed (see Methods section for more details). This shows that our x-ray streaking method does not limit the time resolution, and a significantly higher resolution could be achieved using significantly shorter pump and probe pulses. Furthermore, (self-)seeded XFELs provide pulses with significantly lower bandwidth values, which will enable x-ray streaking experiments with a temporal resolution down to sub-10 fs resolution. For example, if one employs Fourier transform limited pulses of 4 fs duration, the focal spot becomes smaller (20 × 5 μm^2^) and the calculated best time resolution achievable with the same experimental setup becomes ~7 fs.

The maximum achievable time window is determined by the total number of illuminated zones ${N}_{max}$, the wavelength λ of the incoming x-rays, and the speed of light c, according to the relation ${\rm{\Delta }}{t}_{max}={N}_{max}\,\lambda /c.$ For the particular zone plate we used ${\rm{\Delta }}{t}_{max}$ evaluates to approximately 1.6 ps (see Methods section). Typically ${N}_{max}$ is limited by the minimum structure size that can be fabricated and by the available beam size at the XFEL beamline restricting the number of homogeneously illuminated zones. We envision that the maximum time window could be expanded in different ways. For example the use of different fabrication techniques and base materials^23^ would allow tripling the available time window without changing other experimental parameters. An even better figure can be expected by replacing the transmission zone plate with a reflective one, increasing the number of illuminated zones without further increased lithographical effort^24^.

In summary, we have conceived a novel experimental technique that allows for continuous recording of the dynamics of an ultrafast process with a single x-ray pulse, yet maintaining a very high time resolution. We achieved this by employing an off-axis Fresnel zone plate to generate a continuous array of pulses that probe the sample at consecutive, geometrically defined time delays. In a proof-of-principle experiment we recorded the transient change in reflectivity due to the ultrafast demagnetisation process triggered by a near infrared pulse in a cobalt thin layer, using a single x-ray pulse. The measurements shown here not only demonstrate the power of this novel x-ray streaking technique for the investigation of ultrafast processes in single shot experiments, but also illustrate that within our time resolution of ~120 fs no significant fluctuations of the demagnetisation time could be recorded for subsequent demagnetisation events. It should be emphasized that such measurement would not have been possible using traditional repetitive pump probe schemes.

The experimental method demonstrated here paves the way to a series of novel experiments that will benefit from directly recording the transient properties of a sample using a single pump-probe event. For example, one can envision different experimental geometries (e.g. in transmission instead of reflection) based on x-ray streaking to measure x-ray absorption. This will help shine light on chemical processes such as bond formation or charge transfer reactions in crystalline photochemistry, where it is difficult to repeatedly deliver a fresh sample, prerequisite for repetitive pump-probe measurements. Studies in material science will also benefit from the advantages offered by the x-ray streaking method for example in studying the path through irreversible phase transitions in phase change materials^25^. Finally, another field that should benefit from this x-ray streaking technique is the one of warm dense matter where the required excitation intensities typically lead to permanent damage of the sample.

## Methods

### Experimental setup

The experiments were performed at beamline BL2 of the x-ray free electron laser FLASH^26^. The x-ray wavelength was tuned to 20.8 nm, in order to be resonant to the cobalt M_2,3_ absorption edges. The average pulse intensity was (50 ± 12) μJ and the photon energy bandwidth was 1.5% FWHM. FLASH was operated at a repetition rate of 10 Hz and a fast shutter was used to select single x-ray pulses. To keep the x-ray fluence at the sample position low enough to avoid x-ray induced sample modifications, the incoming x-ray pulse was attenuated with two 423 nm thick Al films (18% overall measured transmission), which were positioned upstream of the zone plate. Due to the bandwidth of the x-ray pulses the focal spot is elliptical and its measured size was 200 × 40 μm^2^. The cobalt sample was excited at normal incidence using 800 nm, linearly polarized pulses delivered by the FLASH pump-probe laser. The pump beam was focused on the sample with a spot size of 500 μm in diameter and the polarisation was set parallel to the x-ray scattering plane. Both the pump and the probe spot size were determined by knife-edge scans. The duration of the infrared pump pulse was about 70 fs FWHM, while the probe pulse was about 100 fs FWHM. Both the reference and reflected x-ray beams were collected using two CCD cameras (PI-MTE, Princeton Instruments). To prevent any scattered infrared light from reaching the CCD sensors, and to match the intensity of the reflected and reference beams, a 150 nm and a 3.2 μm thick aluminium filter were installed in front of the respective cameras. The CCD cameras were positioned to match the dimensions of the beam image on both CCDs within 5 pixels. The magnetisation of the sample was saturated before each pump-probe event with a 40 mT magnetic field pulse applied parallel to the sample surface.

### Zone plate details

The off-axis Fresnel zone plates were made from single crystalline Si <111> membranes with lateral dimensions of 4.8 mm × 4.8 mm (Norcada Inc.). The zone plate pattern consisted of ~23000 zone pairs spanning over the full size of the membrane. The outermost zone had a period of 160 nm and a zone radius of 10.3 mm resulting in a focal length of 80 mm at 20.8 nm wavelength. The pattern was defined with an electron-beam writer (Vistec EBPG-5000+ES) operated at 100 keV and etched into the membrane using reactive ion etching. The depth of the zone structures was 400 nm and the remaining thickness of the support membrane was 200 nm. Using the tabulated optical constants of silicon^27^, one can calculate a first order diffraction efficiency of 13.4%. A characterisation of the zone plates at the VUV beamline of the Swiss Light Source revealed, that the actual diffraction efficiency was about 70% of this value due to imperfection in the zone profile. The horizontal line structures in the camera images shown in Fig. 2(a) and (b) are caused by stitching errors of the electron beam writer. These slight offsets on the order of some tens of nanometres cause phase discontinuities in the diffracted wavefronts, which propagate into substantial intensity variations at the camera positions. However, these structures were largely cancelled out by the applied normalization procedure and did not affect the measurements.

### Sample Fabrication

We employed a Co (20 nm) thin layer that was deposited by magnetron sputtering on a 525 μm thick silicon wafer. The cobalt layer is in-plane magnetized and is isotropic in the plane as confirmed by magneto-optic Kerr effect measurements. To ensure optimal growth, a 30 nm thick Pd buffer layer was grown prior to the Co deposition. The layer stack was terminated with a 3 nm thick Al capping layer to prevent oxidation.

### Data Analysis

The ray-optics calculations were performed propagating a set of 247 × 247 rays from the zoneplate surface to the focus. The coordinates were then used to calculate the optical path of light travelling along each ray using the Euclidean distance. These calculations generated the time-delay maps used for the simulation in Fig. 1. The TR-images were extracted from the raw images as described in the text after running an image registration algorithm to minimize the effect of drifts and alignment imperfections. Input from the ray-optics calculation was used to calibrate the time axis of the image and extract the time-resolved reflectivity curve as described in the text. The curves obtained from the TR-images were then fitted by the double exponential expression:

2 $$\frac{{\rm{\Delta }}R}{R}(t)=G(t)\otimes (H(t)({K}_{1}(1-{e}^{\frac{-t}{{\tau }_{s}}}){e}^{\frac{-t}{{\tau }_{s-ph}}}+{K}_{2}(1-{e}^{\frac{-t}{{\tau }_{s-ph}}})))$$

where G(t) is a Gaussian function to take into account the time resolution of the experiment, H(t) is the Heaviside function and ${\tau }_{s}$ and ${\tau }_{s-ph}$ are, respectively, the thermalization time and relaxation time of the spins to other degrees of freedom. This model ignores interaction in the lattice, which are expected to be relevant only on longer time scales. For the data fitting the FWHM of G(t) was set to 120 fs, which corresponds to the convolution of pump and probe pulse duration. Due to the limited time window probed in our experiment, the value of ${\tau }_{s-ph}$ cannot be evaluated reliably from the single shot data. We therefore used a value of 5 ps, which was retrieved from repetitive pump-probe measurements.

### Effect of x-ray bandwidth on time resolution

To simulate the effect of the incoming beam bandwidth on the maximum achievable time resolution we calculated a TR-image generated by a polychromatic beam using a 10 fs FWHM Gaussian time profile as a test function. This was obtained as a weighted average of TR-images generated by the different photon energies contained in a Gaussian spectrum with a bandwidth of 1.5% FWHM. The final TR-image was then processed as the experimental data and the retrieved Gaussian profile showed a FWHM of 51 fs indicating that the maximum possible time resolution for 1.5% bandwidth in this experimental geometry is approximately 50 fs.

### Theoretical modelling

We obtained the time and depth resolved profiles of the transient magnetisation $M(z,t)$ of the sample, by employing the super-diffusive transport model, which treats the propagation of optically excited hot electrons as well as secondary excited electrons through the metal film semi-classically^28^. Since the lifetimes and velocities of the excited electrons are energy and spin-dependent in the magnetic metallic layers, their overall motion leads to a more efficient transport of majority-spin electrons, generating thus a current with a net spin-polarization. The time-dependent dynamics of the spin transport is calculated by numerically solving the super-diffusion equation

3 $$\frac{\partial n(\sigma ,E,z,t)}{\partial t}+\frac{n(\sigma ,E,z,t)}{\tau (\sigma ,E,z)}=(\frac{-\partial }{\partial z}\hat{\Phi }+\hat{I})\times (\hat{S}n(\sigma ,E,z,t)+{S}^{ext}(\sigma ,E,z,t))$$

where $n(\sigma ,E,z,t)$ is the spin- and energy-dependent density of laser-excited electrons, $\tau (\sigma ,E,z)$ is their lifetime, $\hat{\Phi }$ and $\hat{I}$ are the electron flux and identity operators. $\hat{S}$ is an integral operator that computes the source term for the next-generation of electrons, produced through scattering events which result from elastic, inelastic, as well as cascade processes, and ${S}^{ext}(\sigma ,E,z,t)$ is the source term containing explicitly the 70 fs IR pump pulse. The *z* coordinate is defined as being normal to the sample surface. The spin- and excitation-energy-dependent lifetimes and velocities^29, 30^ as well as the ratio of excited majority to minority spin electrons, are taken from *ab initio* calculations. Partial reflection at the interfaces between two layers has been included in the calculations. The energy- and spin-dependent reflectivity has been computed assuming that the electrons cross the interface as classical particles, with velocities defined by the band structure of each material. All possible multiple reflection paths are taken rigorously into account. The simulated system is Al (3 nm)/Co (20 nm)/Pd (30 nm) as specified in the Sample Fabrication section.

### Transient reflectivity calculation

To extract the change in the sample reflectivity due to the ultrafast demagnetisation process and compare it with our experimental results, the depth-resolved transient magnetisation profiles were then used as an input to calculate the expected change in reflectivity due to the T-MOKE effect using the Zak formalism^31^. The optical constants needed for this calculation were retrieved from the Henke tables^27^ for aluminium and palladium, while the one for cobalt were measured directly to guarantee higher accuracy. The transient reflectivity curve obtained using this method was then convoluted with a Gaussian pulse to account for the experimental time resolution.

### Data Availability

The datasets generated during the current study are available from the corresponding author on reasonable request.
